# Mesoscale Dzyaloshinskii-Moriya interaction: geometrical tailoring of the magnetochirality

**DOI:** 10.1038/s41598-017-18835-4

**Published:** 2018-01-16

**Authors:** Oleksii M. Volkov, Denis D. Sheka, Yuri Gaididei, Volodymyr P. Kravchuk, Ulrich K. Rößler, Jürgen Fassbender, Denys Makarov

**Affiliations:** 1 Helmholtz-Zentrum Dresden - Rossendorf e. V., Institute of Ion Beam Physics and Materials Research, 01328 Dresden, Germany; 2 Bogolyubov Institute for Theoretical Physics of the National Academy of Sciences of Ukraine, 03680 Kyiv, Ukraine; 3 Taras Shevchenko National University of Kyiv, 01601 Kyiv, Ukraine; 4 Leibniz-Institut für Festkörper- und Werkstoffforschung (IFW Dresden), 01069 Dresden, Germany

## Abstract

Crystals with broken inversion symmetry can host fundamentally appealing and technologically relevant periodical or localized chiral magnetic textures. The type of the texture as well as its magnetochiral properties are determined by the intrinsic Dzyaloshinskii-Moriya interaction (DMI), which is a material property and can hardly be changed. Here we put forth a method to create new artificial chiral nanoscale objects with tunable magnetochiral properties from standard magnetic materials by using geometrical manipulations. We introduce a mesoscale Dzyaloshinskii-Moriya interaction that combines the intrinsic spin-orbit and extrinsic curvature-driven DMI terms and depends both on the material and geometrical parameters. The vector of the mesoscale DMI determines magnetochiral properties of any curved magnetic system with broken inversion symmetry. The strength and orientation of this vector can be changed by properly choosing the geometry. For a specific example of nanosized magnetic helix, the same material system with different geometrical parameters can acquire one of three zero-temperature magnetic phases, namely, phase with a quasitangential magnetization state, phase with a periodical state and one intermediate phase with a periodical domain wall state. Our approach paves the way towards the realization of a new class of nanoscale spintronic and spinorbitronic devices with the geometrically tunable magnetochirality.

## Introduction

A broken chiral symmetry in a magnetic system manifests itself as the appearance of chiral either periodical (e.g. helical or cycloid modulations^1–4^) or localized magnetization structures (e.g. chiral domain walls^5–7^ and skyrmions^8–14^). The type and magnetic symmetry of this structures are determined by the orientation and strength of the vector of Dzyaloshinskii-Moriya interaction (DMI), which comes from the spin-orbit-driven DMI in bulk magnetic crystals with low symmetry^15,16^ or at interfaces between a ferromagnet and a nonmagnetic material with strong spin-orbit coupling^17–20^. This DMI is *intrinsic* to the crystal or layer stack and, for the case of simplicity, we refer to it as intrinsic DMI (*i*DMI). Recently, it was reported that geometrically-broken symmetry in curvilinear magnetic systems leads to the appearance of *exchange*-*driven DMI*-*like chiral contribution* in the energy functional^21–23^. This chiral term is determined by the sample geometry, e.g. local curvature and torsion, and is therefore *extrinsic* to the crystal or layer stack (*e*DMI). It reveals itself in the domain wall pinning at a localized wire bend^24^ and is responsible for the existence of magnetochiral effects in curvilinear magnetic systems^25^, e.g. coupling of chiralities in spin and physical spaces for the Möbius ring^26^, negative domain wall mobility for helical wires^27–29^.

The magnetic textures of *curvilinear* magnets with *i*DMI will be necessarily determined by the interplay of two types of chiral interactions which are acting at different lengthscales. Hence, in the following we refer to the resulting chiral term of such type as a *mesoscale DMI* (*m*DMI). The symmetry and strength of this term are determined by the geometrical and material properties of a three-dimensional (3D) object. Combining the two DMI offers exciting possibility in tuning the resulting *m*DMI vector. As a consequence, the same material system with properly adjusted geometry can reveals distinct magnetic states.

Here, we study the *m*DMI in a one-dimensional (1D) curvilinear wire. We derive a general expression for the *m*DMI term and analyse the magnetization states which arise in a helix wire. The clear cut comparison with a straight wire with homogeneous *i*DMI reveals: (i) The magnetic states of a curved wire is governed by a single vector *m*DMI, originating from the vector sum of the intrinsic and extrinsic DMI vectors. This provides a possibility to tailor the orientation of the vector of *m*DMI; (ii) The symmetry and period of the chiral structures are determined by the strength and direction of the vector of *m*DMI. Beside the fundamental interest for the community, working with helimagnetic materials, based on our theoretical framework we proposed two new statical methods for determining the intrinsic DMI constant, which is relevant for experimental and material science community.

First, we consider a general case of an arbitrary curved wire, whose circular cross-section diameter is smaller than the characteristic magnetic length, and scrutinize the properties of a curvilinear anisotropic 1D Heisenberg magnet with intrinsic chiral term. In this case the total energy *E* = *E*_an_ + *E*_ex_ + *E*_DMI_ consists of three parts: exchange, anisotropic and *i*DMI contributions, respectively. The transition to the orthogonal curvilinear reference frame allows to get rid of the coordinate dependence of the magnetic anisotropy term *E*_an_. Furthermore, the geometrically broken symmetry leads to the restructuring of all magnetic energy terms containing spatial derivatives. A characteristic example is the transformation of the exchange term into three components ${E}_{{\rm{ex}}}={E}_{{\rm{ex}}}^{0}+{E}_{{\rm{ex}}}^{{\rm{D}}}+{E}_{{\rm{ex}}}^{{\rm{A}}}$ with different symmetry^22^, namely: isotropic part ${E}_{{\rm{ex}}}^{0}$, which has formally the same form as for a straight wire; chiral part ${E}_{{\rm{ex}}}^{{\rm{D}}}$, which represents the geometrically-induced magnetic asymmetry of a wire and plays the role of the curvature-induced DMI; and anisotropic part ${E}_{{\rm{ex}}}^{{\rm{A}}}$, which represents the geometrically-induced magnetic anisotropy of a wire driven by exchange interaction. It should be noted that this chiral and anisotropic terms are sources of emergent “vector” and “scalar” potentials, respectively, for spin-waves in a curved wire^27^. Similar effects in curvature-induced geometrical potential are known from curvilinear quantum-mechanics systems^30^.

The specificity of our case is the presence of a spin-orbit-driven chiral term. In general case of an arbitrary 1D magnetic system the *i*DMI has the following form

1 $${E}_{{\rm{D}}{\rm{M}}{\rm{I}}}=-S\,\int \,{\rm{d}}s\,{{\bf{D}}}^{{\rm{I}}}\cdot [{\bf{m}}\times {{\rm{\partial }}}_{s}{\bf{m}}],$$

see supplementary materials (S6) for details. In (1) *S* is the wire cross-section area; *s* and ∂_*s*_ are arc length of the central line of the wire and the derivative with respect to *s*, respectively; ***D***^I^ is the vector of the *i*DMI; ***m*** is the magnetization unit vector ***m*** = ***M***/*M*_*s*_ with *M*_*s*_ being the saturation magnetization. As the expression (1) contains the coordinate derivative, the transition to the orthogonal curvilinear reference frame results in appearance of effective curvature-induced anisotropy^31^. Physically this means that the *i*DMI will necessarily contribute both to chiral and anisotropy terms. By performing the transition to the curvilinear Frenet-Serret (TNB) reference frame {***e***_T_, ***e***_N_, ***e***_B_}, with ***e***_T_ being a tangential, ***e***_N_ being a normal and ***e***_B_ being a binormal vector and following the approach^22^, we group al terms of the total energy in three categories containing isotropic exchange, chiral and anisotropic parts:

2a $$E=KS\,\int \,\,{\rm{d}}\xi \,[{m^{\prime} }_{\alpha }\,{m^{\prime} }_{\alpha }+{{\mathcal{D}}}_{\alpha \beta }^{{\rm{meso}}}({m}_{\alpha }\,{m^{\prime} }_{\beta }-{m^{\prime} }_{\alpha }\,{m}_{\beta })+{{\mathcal{K}}}_{\alpha \beta }^{{\rm{meso}}}\,{m}_{\alpha }{m}_{\beta }],$$

2b $$\parallel {\mathcal{D}}_{\alpha \beta }^{{\rm{m}}{\rm{e}}{\rm{s}}{\rm{o}}}\parallel =\parallel \begin{array}{ccc}0 & -({{\mathcal{D}}}_{{\rm{B}}}^{{\rm{I}}}-2\varkappa )/2 & -{{\mathcal{D}}}_{{\rm{N}}}^{{\rm{I}}}/2\\ ({{\mathcal{D}}}_{{\rm{B}}}^{{\rm{I}}}-2\varkappa )/2 & 0 & -({{\mathcal{D}}}_{{\rm{T}}}^{{\rm{I}}}-2\sigma )/2\\ {{\mathcal{D}}}_{{\rm{N}}}^{{\rm{I}}}/2 & ({{\mathcal{D}}}_{{\rm{T}}}^{{\rm{I}}}-2\sigma )/2 & 0\end{array}\parallel ,$$

2c $$\parallel {\mathcal{K}}_{\alpha \beta }^{{\rm{m}}{\rm{e}}{\rm{s}}{\rm{o}}}\parallel =\parallel \begin{array}{ccc}\sigma ({{\mathcal{D}}}_{{\rm{T}}}^{{\rm{I}}}-\sigma )-1 & {{\mathcal{D}}}_{{\rm{N}}}^{{\rm{I}}}\sigma /2 & (\varkappa {{\mathcal{D}}}_{{\rm{T}}}^{{\rm{I}}}+\sigma {{\mathcal{D}}}_{{\rm{B}}}^{{\rm{I}}}-2\varkappa \sigma )/2\\ {{\mathcal{D}}}_{{\rm{N}}}^{{\rm{I}}}\sigma /2 & 0 & {{\mathcal{D}}}_{{\rm{N}}}^{{\rm{I}}}\varkappa /2\\ (\varkappa \,{{\mathcal{D}}}_{{\rm{T}}}^{{\rm{I}}}+\sigma {{\mathcal{D}}}_{{\rm{B}}}^{{\rm{I}}}-2\varkappa \sigma )/2 & {{\mathcal{D}}}_{{\rm{N}}}^{{\rm{I}}}\varkappa /2 & \varkappa \,({{\mathcal{D}}}_{{\rm{B}}}^{{\rm{I}}}-\varkappa ),\end{array}\parallel $$

which is the general expression and it is valid for any *i*DMI and wire geometry, that can include possible coordinate dependence ${\boldsymbol{\mathcal{D}}}$^I^(*s*), $\varkappa \,(s)$, and *σ*(*s*). In the expression (2) $K={K}_{0}+\pi {M}_{s}^{2}$ is the effective anisotropy constant, with *K*_0_ > 0 being the magnetocrystalline anisotropy of easy-tangential type. The term $\pi {M}_{s}^{2}$ is the effective local anisotropy constant caused by surface magnetostatic charges: in the main approach on a thickness of a curved wire the non-local magnetostatic interaction is rigorously shown^32^ to be reduced to the effective local anisotropy. The prime denotes the derivative with respect to the dimensionless coordinate *ξ* = *s*/*w*, where $w=\sqrt{A/K}$ is the characteristic magnetic length, with *A* being an exchange constant and the Einstein summation rule is applied on Greek indices *α*, *β* = T, N, B. In (2) ${{\mathcal{D}}}_{\alpha \beta }^{{\rm{meso}}}$ and ${{\mathcal{K}}}_{\alpha \beta }^{{\rm{meso}}}$ are *m*DMI and anisotropy parameters, respectively; ${{\boldsymbol{D}}}^{I}=({{\mathcal{D}}}_{{\rm{T}}}^{{\rm{I}}},{{\mathcal{D}}}_{{\rm{N}}}^{{\rm{I}}},{{\mathcal{D}}}_{{\rm{B}}}^{{\rm{I}}})={{\boldsymbol{\mathcal{D}}}}^{{\rm{I}}}/\sqrt{A\,K}$ being the reduced vector of the *i*DMI. One can also introduce a vector of *e*DMI for curvilinear wires ${{\boldsymbol{\mathcal{D}}}}^{{\rm{E}}}=(-2\sigma ,\,0,-2\varkappa )$, where $\varkappa (\xi )=w\,\kappa \,(\xi )$ and *σ*(*ξ*) = *wτ*(*ξ*) are the reduced local curvature and torsion of the wire, with *κ*(*ξ*) and *τ*(*ξ*) being the local curvature and torsion, respectively. For the case of planar curvilinear wire (*τ* = 0), the vector of the exchange-driven DMI is always perpendicular to the wire plane^22,27^. It should be noted, that anisotropy constants linear in the curvature and in the torsion are generated by the *i*DMI, while quadratic terms come from the exchange interaction.

Remarkably, the *m*DMI term contains the full set of Lifshitz invariants in the TNB reference frame. Therefore, it is instructive to introduce the vector of the *m*DMI

3 $${\bf{D}}={{\bf{D}}}^{{\rm{I}}}+{{\bf{D}}}^{{\rm{E}}}=({{\mathcal{D}}}_{{\rm{T}}}^{{\rm{I}}}-2\sigma )\,{{\bf{e}}}_{{\rm{T}}}+{{\mathcal{D}}}_{{\rm{N}}}^{{\rm{I}}}\,{{\bf{e}}}_{{\rm{N}}}+({{\mathcal{D}}}_{{\rm{B}}}^{{\rm{I}}}-2\varkappa )\,{{\bf{e}}}_{{\rm{B}}},$$

where ${\boldsymbol{\mathcal{D}}}$ is a vector sum of the DMI vectors of the intrinsic and the extrinsic types, respectively. Thus, for a curved 1D object, the vector ${\boldsymbol{\mathcal{D}}}$ determines a new direction of effective DMI in the system. It should be emphasized that ${\boldsymbol{\mathcal{D}}}$ is dependent on both geometrical and material properties of the sample.

**Figure 1:**
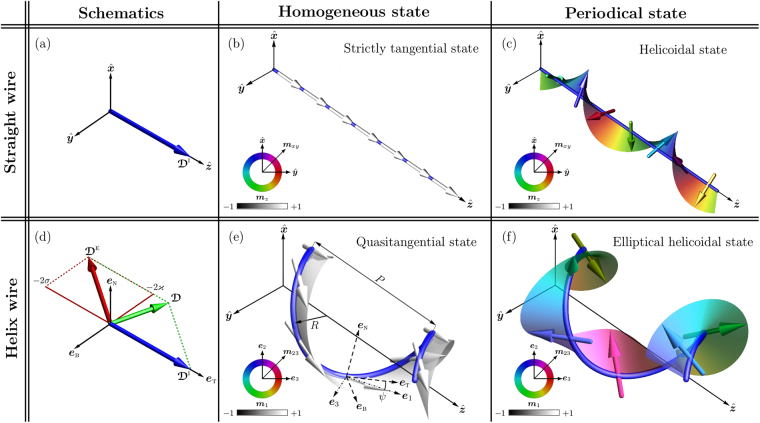
Schematic illustration of the interplay between the intrinsic and extrinsic DMI and the resulting magnetization distributions in a wire. (**a**) The vector of *i*DMI in the Cartesian frame of reference for a straight wire. (**b**,**c**) Tangential homogeneous ($\varkappa =0$, *σ* = 0, ${{\mathcal{D}}}_{{\rm{T}}}^{{\rm{I}}}=0$) and periodical helicoidal ($\varkappa =0$, *σ* = 0, ${{\mathcal{D}}}_{{\rm{T}}}^{{\rm{I}}}=2.7$) states in a straight wire with the easy-tangential anisotropy and *i*DMI. (d) Vectors of the *i*DMI and *e*DMI in the TNB reference frame. (**e**,**f**) Quasitangential ($\varkappa =0.8$, *σ* = 0.5, ${{\mathcal{D}}}_{{\rm{T}}}^{{\rm{I}}}=0$, ${\mathcal{C}}=+1$) and periodical ($\varkappa =0.8$, *σ* = 0.5, ${{\mathcal{D}}}_{{\rm{T}}}^{{\rm{I}}}=2.7$, ${\mathcal{C}}=+1$) states in a helical wire with the easy-tangential anisotropy and *m*DMI obtained from numerical simulations (see Methods). Color arrows correspond to the magnetic moments. The Cartesian, the Frenet-Serret {***e***_T_, ***e***_N_, ***e***_B_} and the rotated {***e***_1_, ***e***_2_, ***e***_3_} reference frames are shown with solid, dashed and dashed-dot lines, respectively in (**e**).

In the following we apply the general approach of *m*DMI to the specific example of a helical wire with *i*DMI and compare our results with the case of a straight wire with same *i*DMI. Helix is the simplest curvilinear system with both curvature and torsion, which has the following parametrization: ${\boldsymbol{\gamma }}(s)=\hat{{\boldsymbol{x}}}\,R\,\cos (s/{s}_{0})+\hat{{\boldsymbol{y}}}\,R\,\sin (s/{s}_{0})+\hat{{\boldsymbol{z}}}\,{\mathcal{C}}Ps/(2\pi {s}_{0})$. Here *R* is the helix radius, *P* is the pitch of the helix, ${\mathcal{C}}=\pm 1$ is the helix chirality, namely, ${\mathcal{C}}=-1$ for the clockwise (right) helix and ${\mathcal{C}}=1$ for the counterclockwise (left) one and ${s}_{0}=\sqrt{{R}^{2}+{P}^{2}/{\mathrm{(2}\pi )}^{2}}$. Helix is characterized by the constant curvature $\kappa =R/{s}_{0}^{2}$ and torsion $\tau ={\mathcal{C}}P/\mathrm{(2}\pi {s}_{0}^{2})$. It should be noted, that for such kind of curvature and torsion definition, *κ* is always positive, while sign of *τ* is determined by the helix chirality. We consider a homogeneous *i*DMI with a vector, which is aligned along the tangential direction of a wire, namely: in the case of a straight wire ${{\boldsymbol{\mathcal{D}}}}^{{\rm{I}}}={{\mathcal{D}}}_{{\rm{T}}}^{{\rm{I}}}\,\hat{{\boldsymbol{z}}}$, which is similar to those in a cubic noncentrosymmetric magnets^33^, Fig. 1(a); in the case of a helical wire ${{\boldsymbol{\mathcal{D}}}}^{{\rm{I}}}={{\mathcal{D}}}_{{\rm{T}}}^{{\rm{I}}}\,{{\boldsymbol{e}}}_{{\rm{T}}}$, while the vector of the *m*DMI ${\boldsymbol{\mathcal{D}}}=({{\mathcal{D}}}_{{\rm{T}}}^{{\rm{I}}}-2\sigma )\,{{\boldsymbol{e}}}_{{\rm{T}}}-2\varkappa \,{{\boldsymbol{e}}}_{{\rm{B}}}$ lies in the TB-plane, Fig. 1(d). Should be indicated, that for the specific value of the torsion $\sigma ={{\mathcal{D}}}_{{\rm{T}}}^{{\rm{I}}}/2$ the direction of the *m*DMI vector becomes perpendicular to the initial tangential direction, which can be interpreted as a change of type of the DMI from the bulk one to the interfacial one.

In the case of a weak *i*DMI (for the case of a straight wire) or *m*DMI (for the case of a helical wire), in both magnetic systems homogeneous magnetization ground state appears: For a straight wire, the magnetization is aligned strictly along the wire (tangential direction), Fig. 1(b). For a helical wire, the existence of the exchange- and the *i*DMI-induced anisotropies in (2) prevents the appearance of the equilibrium homogeneous tangential state, Fig. 1(e). As a result, magnetization vectors are tilted by a constant angle *ψ* from the tangential direction ***e***_T_:

4 $$\psi \approx \varkappa \,(\sigma -{{\mathcal{D}}}_{{\rm{T}}}^{{\rm{I}}}/2),\,{\rm{w}}{\rm{i}}{\rm{t}}{\rm{h}}\,\varkappa ,|\sigma -{{\mathcal{D}}}_{{\rm{T}}}^{{\rm{I}}}/2|\ll 1.$$

Therefore, this state is referred to as the homogeneous (in the curvilinear reference frame) *quasitangential* state, Fig. 1(e). We note that even in the presence of a strong easy-axis anisotropy and absence of the *i*DMI (${{\mathcal{D}}}_{{\rm{T}}}^{{\rm{I}}}=0$), the resulting effective anisotropy in 1D wire is biaxial^29^. It is instructive to mention a specific case $\sigma ={\sigma }_{0}={{\mathcal{D}}}_{{\rm{T}}}^{{\rm{I}}}/2:$ the interplay between the exchange- and *i*DMI-driven anisotropies results in a strictly tangential state (but anisotropy remains biaxial).

By rotating the reference frame by the angle *ψ* we diagonalize the effective mesoscopic anisotropy tensor of a helical wire (2). For the case of simplicity, it is useful to make transition to the new frame of reference by making rotation by the angle *ψ* around the axis ***e***_N_ in the positive direction. In the rotated *ψ*–frame {***e***_1_, ***e***_2_, ***e***_3_} [Fig. 1(e)] with the magnetization $\mathop{{\bf{m}}}\limits^{ \sim }={m}_{1}{{\bf{e}}}_{1}+{m}_{2}{{\bf{e}}}_{2}+{m}_{3}{{\bf{e}}}_{3}$ the energy density has the following form:

5 $$ {\mathcal E} =|\tilde{{\boldsymbol{m}}}^{\prime} {|}^{2}-{{\mathcal{K}}}_{1}{m}_{1}^{2}+{{\mathcal{K}}}_{2}{m}_{2}^{2}+{{\mathcal{D}}}_{1}({m}_{2}{m^{\prime} }_{3}-{m}_{3}{m^{\prime} }_{2})+{{\mathcal{D}}}_{2}({m}_{1}{m^{\prime} }_{2}-{m}_{2}{m^{\prime} }_{1}\mathrm{)}.$$

While the coefficient ${{\mathcal{K}}}_{1} > 0$ characterizes the strength of the effective easy-axis anisotropy, the ${{\mathcal{K}}}_{2} > 0$ gives the strength of the effective hard-axis anisotropy. The parameters ${{\mathcal{D}}}_{1}$ and ${{\mathcal{D}}}_{2}$ are the effective *m*DMI constants, which are responsible for two types of the magnetization rotation: around the direction ***e***_1_ and ***e***_3_, respectively. The exact expressions for ${{\mathcal{K}}}_{i}$ and ${{\mathcal{D}}}_{i}$ with *i* = 1, 2 are given in supplementary materials. In *ψ*–frame, the quasitangential state becomes aligned along the direction ***e***_1_, Fig. 1(e) (dash-dotted line), and its energy density reads ${ {\mathcal E} }^{{\rm{qt}}}=-{{\mathcal{K}}}_{1}$.

**Figure 2:**
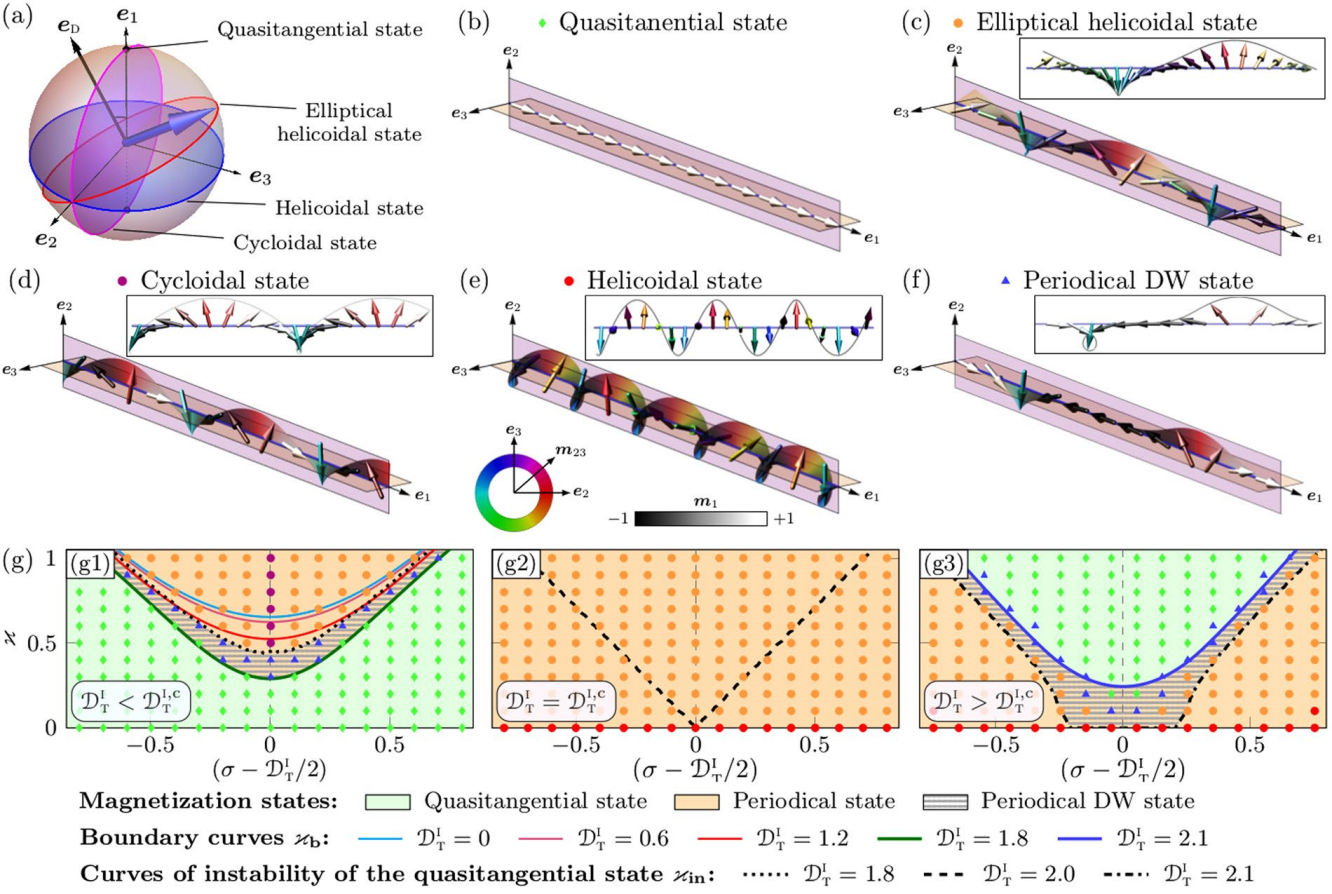
Phase diagrams of the equilibrium magnetization states for helical wires with different geometrical and material properties. (**a**) The evolution of the magnetization vector $\tilde{{\boldsymbol{m}}}(\xi )$ on a unit sphere for different magnetization states in a helical wire. (**b**–**f**) Schematics of the equilibrium magnetization states which appear for different values of the *m*DMI in the rotated *ψ*–frame {***e***_1_, ***e***_2_, ***e***_3_}: quasitangential state ($\varkappa =0.8$, *σ* = 0.5, ${{\mathcal{D}}}_{{\rm{T}}}^{{\rm{I}}}=0$, ${\mathcal{C}}=+1$), elliptical helicoidal state ($\varkappa =0.8$, *σ* = 0.5, ${{\mathcal{D}}}_{{\rm{T}}}^{{\rm{I}}}=1.2$, ${\mathcal{C}}=+1$), cycloidal state ($\varkappa =0.9$, *σ* = 0.6, ${{\mathcal{D}}}_{{\rm{T}}}^{{\rm{I}}}=1.2$, ${\mathcal{C}}=+1$), helicoidal state ($\varkappa =0.1$, *σ* = 0.6, ${{\mathcal{D}}}_{{\rm{T}}}^{{\rm{I}}}=2.1$, ${\mathcal{C}}=-1$) and periodical DW state ($\varkappa =0.7$, *σ* = 0.3, ${{\mathcal{D}}}_{{\rm{T}}}^{{\rm{I}}}=0.6$, ${\mathcal{C}}=+1$). (**g**) Phase diagrams of the equilibrium magnetization states for helical wires with different geometrical parameters (reduced curvature $\varkappa$ and torsion *σ*) and *i*DMI with different strength ${{\mathcal{D}}}_{{\rm{T}}}^{{\rm{I}}}$. (g1–g3) Phase diagrams for ${{\mathcal{D}}}_{{\rm{T}}}^{{\rm{I}}}=1.8$, ${{\mathcal{D}}}_{{\rm{T}}}^{{\rm{I}}}=2.0$ and ${{\mathcal{D}}}_{{\rm{T}}}^{{\rm{I}}}=2.1$, respectively. Symbols correspond to the results of numerical simulations: green diamonds represent the homogeneous quasitangential state; purple, red and orange circles indicate to the cycloidal, helicoidal and elliptical helicoidal states, respectively; shaded region correspond to the periodical DW state.

In the case of a sufficiently strong *i*DMI or/and big values of the reduced curvature and torsion, in both magnetic systems appear periodical states, Fig. 1(c,f). Contrary to the case of a straight wire where the symmetry and period of the chiral modulation are defined by the direction and strength of the *i*DMI vector ${{\boldsymbol{\mathcal{D}}}}^{{\rm{I}}}$, in the case of a helical wire, the chiral modulations are dependent on the direction and strength of the vector of *m*DMI ${\bf{D}}$. This means that a curvilinear system is characterized by both spin-orbit and spin-geometry couplings, which magnetochiral properties are determined by *m*DMI vector ${\bf{D}}$. Hence, while in the case of a straight wire, chiral modulations form a helicoidal state^1,2,33^, Fig. 1(c), the general form of the periodical structure in a helical wire is an *elliptical helicoidal state*, Fig. 1(f). Using the angular parametrization $\mathop{{\bf{m}}}\limits^{ \sim }=\,\cos \theta \,{{\boldsymbol{e}}}_{1}+\,\sin \theta \,\cos \varphi \,{{\boldsymbol{e}}}_{2}+\,\sin \theta \,\sin \varphi \,{{\bf{e}}}_{3}$ for the energy functional (5) and the Landau-Lifshitz equation (S2), we obtain

6 $$\begin{array}{ccc}\sin \theta \,\dot{\varphi } & = & -\theta {\rm{^{\prime} }}{\rm{^{\prime} }}+\frac{1}{2}\,\sin 2\theta \,[{(\varphi {\rm{^{\prime} }})}^{2}+{{\mathcal{K}}}_{1}+{{\mathcal{K}}}_{2}\,{\cos }^{2}\varphi +{{\mathcal{D}}}_{1}\varphi {\rm{^{\prime} }}]+{{\mathcal{D}}}_{2}\,{\sin }^{2}\theta \,\sin \varphi \,\varphi {\rm{^{\prime} }},\\ \sin \theta \,\dot{\theta } & = & \frac{1}{2}\,\sin 2\theta \,[2\,\theta {\rm{^{\prime} }}\varphi {\rm{^{\prime} }}+{{\mathcal{D}}}_{1}\theta {\rm{^{\prime} }}]+\frac{1}{2}\,{\sin }^{2}\theta \,[2\varphi {\rm{^{\prime} }}{\rm{^{\prime} }}+{{\mathcal{K}}}_{2}\,\sin 2\varphi +2{{\mathcal{D}}}_{2}\,\sin \varphi \,\theta {\rm{^{\prime} }}],\end{array}$$

where the overdot indicates a derivative with respect to rescaled time which is measured in units of (2*γ*_0_*K*/*M*_*s*_)^−1^, where *γ*_0_ is a gyromagnetic ratio. The stationary solution for the periodical state in the rotated *ψ*-frame can be presented in the following form,

7 $${\theta }^{{\rm{p}}{\rm{e}}{\rm{r}}}(\xi )=\frac{\pi }{2}+\vartheta (\frac{{\mathcal{D}}}{2}\xi ),\quad {\varphi }^{{\rm{p}}{\rm{e}}{\rm{r}}}(\xi )=-\frac{{\mathcal{D}}}{2}\xi +\phi (\frac{{\mathcal{D}}}{2}\xi ),$$

where *ϑ*(*χ*) and *φ*(*χ*) are the 2*π*–periodical functions, with $$\chi ={\mathcal{D}}\,\xi /2$$ and $${\mathcal{D}}=\sqrt{{{\mathcal{D}}}_{1}^{2}+{{\mathcal{D}}}_{2}^{2}}=$$$$\sqrt{{({{\mathcal{D}}}_{{\rm{T}}}^{{\rm{I}}}-2\sigma )}^{2}+4{\varkappa }^{2}}$$ being the strength of the *m*DMI. When the *i*DMI is absent ($${{\mathcal{D}}}_{{\rm{T}}}^{{\rm{I}}}=0$$) the period of the periodical state matches with the geometrical period of the helical wire^27^. Hence, the value of $${\mathcal{D}}$$ determines the period of the chiral modulations, while the direction $${{\bf{e}}}_{{\rm{D}}}={\boldsymbol{\mathcal{D}}}/{\mathcal{D}}$$ determines their symmetry, Fig. 2(a). By tuning $${{\bf{D}}}^{{\rm{I}}}$$ and $${{\bf{D}}}^{{\rm{E}}}$$ it is possible to rotate ***e***_D_ at will, e.g. to obtain two specific states characteristic to a straight wire with different symmetry of *i*DMI^33^:

(i) *Cycloidal state*, which appears in the *ψ*-frame of references for ${{\mathcal{D}}}_{1}=0$, ${{\mathcal{D}}}_{2}\ne 0$ and corresponds to the situation when the magnetization undergoes chiral modulations around ***e***_3_-axis, Fig. 2(d). This case matches the situation when the vector ***e***_D_ is aligned along the hard axis ***e***_3_, Fig. 2(a).
(ii) *Helicoidal state*, which corresponds to the chiral modulation around the axis ***e***_1_ in the *ψ*-frame of references, Fig. 2(e). This case is realized for ${{\mathcal{D}}}_{1}\ne 0$, ${{\mathcal{D}}}_{2}=0$, which corresponds to helicies with large radii and straight wires, and matches the situation, when ***e***_D_ is aligned along the easy axis ***e***_1_, Fig. 2(a).

Expanding the $\vartheta ({\mathcal{D}}\,\xi /2)$ and $\phi ({\mathcal{D}}\,\xi /2)$ into the Fourier series and minimizing the total energy with respect to Fourier amplitudes we derive numerically the energy density of the periodical state ${ {\mathcal E} }_{{\rm{per}}}$. By comparing energies of the quasitangential and periodical states, we determine the boundary curve ${\varkappa }_{{\rm{b}}}={\varkappa }_{{\rm{b}}}(\sigma ,{{\mathcal{D}}}_{{\rm{T}}}^{{\rm{I}}})$, which separates two stable phases under the condition ${E}^{{\rm{q}}{\rm{t}}}(\sigma ,\varkappa ,{{\mathcal{D}}}_{{\rm{T}}}^{{\rm{I}}})={E}^{{\rm{p}}{\rm{e}}{\rm{r}}}(\sigma ,\varkappa ,{{\mathcal{D}}}_{{\rm{T}}}^{{\rm{I}}})$^22^, Fig. 2(g). The quasitangential state can also exist inside the periodical phase as a metastable state and vice versa. Similar scenario was discussed recently in the case of a straight wire with the *i*DMI directed perpendicular to the easy-axis of magnetization^4^ and for the helices with *e*DMI only^27^. The corresponding metastable state forms a *periodical domain wall state*. The instability curve of this state is determined by vanishing the spin-wave spectrum gap on the background of the homogeneous state^27^. The dispersion relation of spin waves on the background of the quasitangential state has the following form

8 $${\rm{\Omega }}(q)=-{{\mathcal{D}}}_{1}q+\sqrt{({q}^{2}+{{\mathcal{K}}}_{1}+{{\mathcal{K}}}_{2})\,({q}^{2}+{{\mathcal{K}}}_{1})}.$$

Here Ω corresponds to the dimensionless eigenfrequency and dimensionless wave number *q* = *kw* with *k* being the wave number, whose wave vector is oriented along the wire. The dispersion law (8) violates the mirror symmetry (frequency nonreciprocity of spin waves) and has a gap, which depends on the *m*DMI constant ${{\mathcal{D}}}_{1}$. This gap vanishes at the critical wave number ${q}_{c}=\sqrt{({{\mathcal{D}}}_{1}^{2}-2{{\mathcal{K}}}_{1}-{{\mathcal{K}}}_{2}\mathrm{)/2}}$ determining the instability curve ${\varkappa }_{{\rm{in}}}$ of the quasitangential state and transition of magnetic system to the continuously periodical state. This instability curves are shown on the phase diagrams Fig. 2(g1–g3) by dotted, dashed and dashdotted curves, respectively. It is important to note that in the case of absence of *i*DMI the dispersion relation (8) makes a transition to the previously obtained one^27^. The region between the boundary and instability curves can have metastable states (shaded area on Fig. 2(g1–g3)), which represent mix of periodical and homogeneous states in the form of periodical domain wall state. In the limit case of straight wire, the instability curve converges to a point ${{\mathcal{D}}}_{{\rm{T}}}^{{\rm{I}},{\rm{c}}}=2$ $({D}_{{\rm{T}}}^{{\rm{I}},{\rm{c}}}=2\sqrt{AK})$, which defines the direct transition from the homogeneously tangential to the periodical state without any metastable states^33^. It should be mentioned that the dispersion relation of a spin-waves on the background of the helicoidal state in the case of straight wire has a band structure^34^.

In general case of presence both kinds of DMI, there are three types of magnetic states in the system: quasitangential state; periodical state and intermediate state with periodical DW. It should be noted, that intermediate periodical DW state appears in any finite systems due to the boundary conditions. The summary of the phase diagrams is presented in Fig. 2(g) with indicated boundary ${\varkappa }_{{\rm{b}}}(\sigma ,{{\mathcal{D}}}_{{\rm{T}}}^{{\rm{I}}})$ and instability ${\varkappa }_{{\rm{i}}{\rm{n}}}(\sigma ,{{\mathcal{D}}}_{{\rm{T}}}^{{\rm{I}}})$ curves for three different values of the *i*DMI: below, equal and above the critical value ${{\mathcal{D}}}_{{\rm{T}}}^{{\rm{I}},{\rm{c}}}$:

- ${{\mathcal{D}}}_{{\rm{T}}}^{{\rm{I}}} < {{\mathcal{D}}}_{{\rm{T}}}^{{\rm{I}},{\rm{c}}}$ [Fig. 2(g1)]: the phase with the periodical state (orange-shaded region) is situated above the phase of the quasitangential state (green-shaded region) and the phase diagram is symmetrical with respect to the line $\sigma ={\sigma }_{0}={{\mathcal{D}}}_{{\rm{T}}}^{{\rm{I}}}/2$. When the value ${{\mathcal{D}}}_{{\rm{T}}}^{{\rm{I}}}$ increases the periodical phase becomes shifted to the range of large positive *σ*, while the boundary curve ${\varkappa }_{{\rm{b}}}(\sigma ,{{\mathcal{D}}}_{{\rm{T}}}^{{\rm{I}}})$ (green solid line) separating the quasitangential and the periodical states approaches the instability curve ${\varkappa }_{{\rm{i}}{\rm{n}}}(\sigma ,{{\mathcal{D}}}_{{\rm{T}}}^{{\rm{I}}})$ (dotted line). In the region between these curves appears periodical domain wall state.
- ${{\mathcal{D}}}_{{\rm{T}}}^{{\rm{I}}}={{\mathcal{D}}}_{{\rm{T}}}^{{\rm{I}},{\rm{c}}}$ [Fig. 2(g2)]: the boundary and instability curves coincide and the elliptical helicoidal state becomes a ground state of the magnetic helix, while for straight wires ($\varkappa =0$) the helicoidal state exists.
- ${{\mathcal{D}}}_{{\rm{T}}}^{{\rm{I}}} > {{\mathcal{D}}}_{{\rm{T}}}^{{\rm{I}},{\rm{c}}}$ [Fig. 2(g3)]: the boundary between the quasitangential and periodical states reappears. However, the interplay between the geometrical and magnetic chiralities leads to the swap of magnetic phases (green-shaded region is at the top of the phase diagram), compare Fig. 2(g1,g3). Further increase of ${{\mathcal{D}}}_{{\rm{T}}}^{{\rm{I}}}$ leads to the shift of the quasitangential phase to the region of large positive values of the reduced torsion *σ* and curvature $\varkappa$.

**Figure 3:**
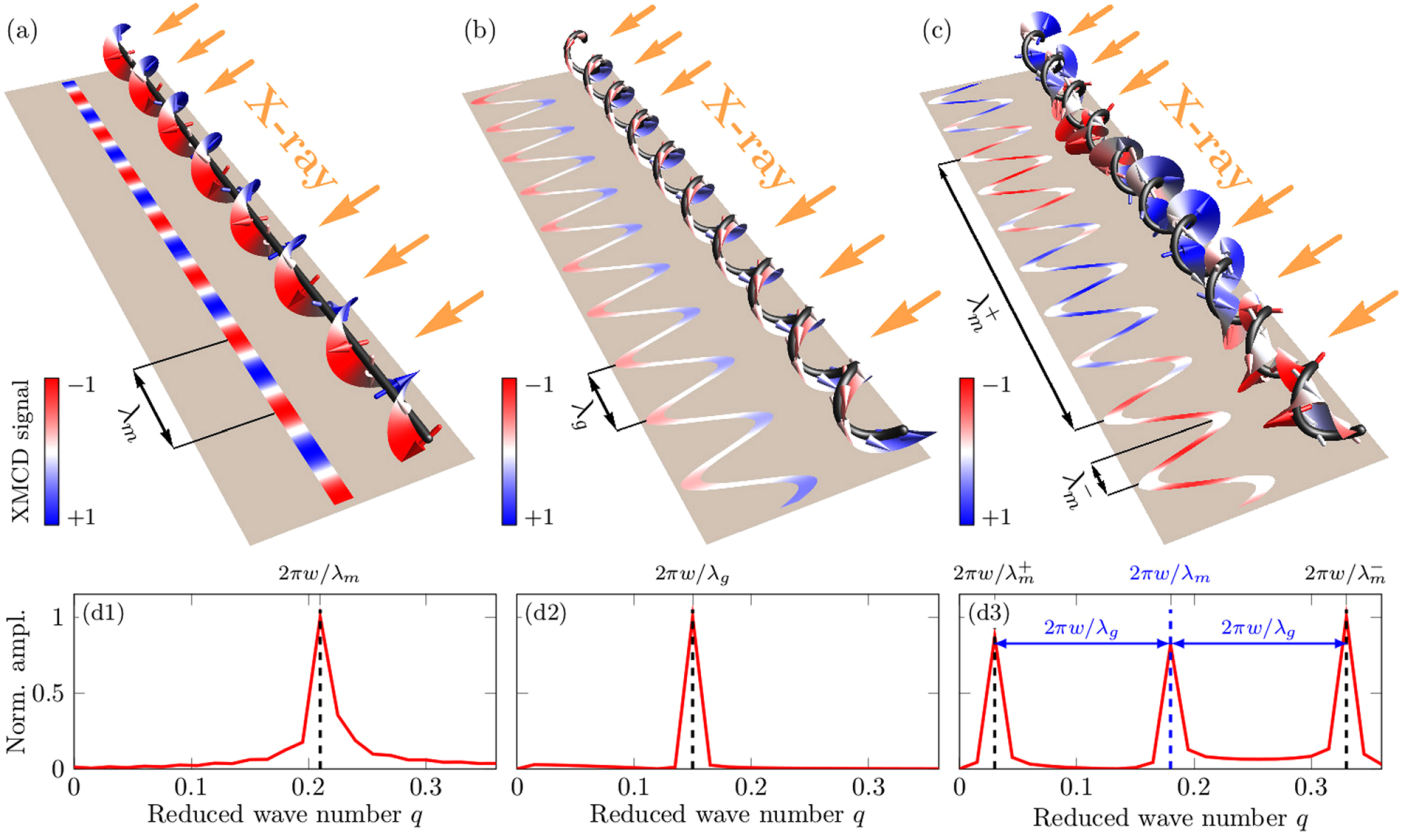
XMCD-PEEM-like numerical experiments with magnetic wires, where the x-ray beam hits the samples under the angle of 25° with respect to the surface plane. (**a**) The helicoidal state in a straight wire with ${{\mathcal{D}}}_{{\rm{T}}}^{{\rm{I}}}=2.7$. (**b**) The quasitangential state in a helical wire with $\varkappa =0.8$, *σ* = 0.5, ${{\mathcal{D}}}_{{\rm{T}}}^{{\rm{I}}}=0$, ${\mathcal{C}}=+1$. (**c**) The periodical state with $\varkappa =0.8$, *σ* = 0.5, ${{\mathcal{D}}}_{{\rm{T}}}^{{\rm{I}}}=2.7$, ${\mathcal{C}}=+1$. Colors of the surface of the magnetization rotation and the XMCD-PEEM-like contrast are equal and reveal the magnetization parallel (red) and antiparallel (blue) to the x-ray beam. (d1–d3) Fourier spectra of the XMCD-PEEM-like signal along the wires for the helicoidal, quasitangential and periodical states, respectively.

In the following we discuss in details the magnetization states which appear in a helical wire due to the influence of the *m*DMI:

(i) The geometrically-induced anisotropy prevents the appearance of the equilibrium strictly tangential state and cause the tilt of the magnetization by the angle *ψ* with respect to the tangential direction. In the case when the *i*DMI is absent (${{\mathcal{D}}}_{{\rm{T}}}^{{\rm{I}}}=0$) the tilt angle *ψ*_−1_ for the clockwise helix (${\mathcal{C}}=-1$) is equal to the tilt angle *ψ*_+1_ for the counterclockwise helix (${\mathcal{C}}=+1$). At the same time, when the *i*DMI is present (${{\mathcal{D}}}_{{\rm{T}}}^{{\rm{I}}}\ne 0$) the tilt angles *ψ*_−1_ and *ψ*_+1_ are different resulting in a different average remanent magnetizations for both types of helices ${\langle {\bf{m}}\rangle }_{\pm 1}=\hat{{\bf{z}}}\,\sin ({\psi }_{\pm 1}+{\alpha }_{\pm 1})$, with ${\alpha }_{\pm 1}=\pm \arctan (|\sigma |/\varkappa )$. Thus, by measuring the ratio between the average remanence for clockwise and counterclockwise helices it is possible to access the value of the *i*DMI:
  9 $${r}_{m}=\frac{|{\langle {\boldsymbol{m}}\rangle }_{+1}|}{|{\langle {\boldsymbol{m}}\rangle }_{-1}|}=|\frac{\varkappa \,\sin \,{\psi }_{+1}+\sigma \,\cos \,{\psi }_{+1}}{\varkappa \,\sin \,{\psi }_{-1}-\sigma \,\cos \,{\psi }_{-1}}|.$$
  For instance, in the case of clockwise and counterclockwise helices with $\varkappa =0.5$, |*σ*| = 0.3, ${{\mathcal{D}}}_{{\rm{T}}}^{{\rm{I}}}=0$ the ratio is equal to 1, while for ${{\mathcal{D}}}_{{\rm{T}}}^{{\rm{I}}}=0.6$ the ratio *r*_*m*_ = 0.71. In the same time, in the case of periodical state, the average remanent magnetization along the helix wire will be absent 〈*m*_*z*_〉 = 0.
(ii) Alternatively, it is possible to access the value for *i*DMI from the analysis of the microscopic images of the periodical magnetic states taken by using microscopic techniques, e.g. Lorentz electron microscopy^35^, electron holography^36^, magnetic transmission X-ray microscope (MTXM)^37,38^ and X-ray magnetic circular dichroism photoelectron emission microscope (XMCD-PEEM). We illustrate this possibility for an exemplarily choosen XMCD-PEEM-like experiment. We note that the XMCD-PEEM was used to study magnetic states in curved architectures with a spatial resolution of 50 nm^39,40^. Still, to apply the present proposal in practical settings, we will necessarily need to work with a helix of several periods. This is required to perform a reliable Fourier analysis. Therefore, realistically, the method might be applied to helices with a length of about 10 *μ*m. The comparison with the case of a straight wire reveals the possibility to distinguish all types of equilibrium magnetization states in helical wires, Fig. 3(a–c). Analysis of the space Fourier spectra of the calculated XMCD-PEEM-like signals allow to determine the magnetic and geometrical periods: in the case of a straight wire, there is only one peak in the Fourier spectrum, which represents the magnetic period of the helicoidal state, *λ*_*m*_, Fig. 3(d1). In the case of a helical wire, the existence of one peak reveals that the magnetic wire is in the homogeneous quasitangential state and the position of the peak provides access to the geometrical period, *λ*_*g*_, of the wire Fig. 3(d2). The existence of three peaks is a clear manifestation of the periodical magnetization state in a helical wire, because of the beating between the geometrical and magnetic modulations of the helical wire, Fig. 3(d3). Meanwhile, the central peak could be used for distinguishing the value of *m*DMI strength ${\mathcal{D}}$.

Accessing spin textures of geometrically curved magnetic thin films^39,41–44^, hollow cylinders^37,40,45–50^ and wires^35,51–54^ has become now a dynamic research field^23,55^. In our work, we performed a detailed study of the interplay between the intrinsic and extrinsic chiral interactions of 3D curvilinear objects within a 1D anisotropic Heisenberg magnet with intrinsic DMI. We established that the chiral properties of the magnetic system are necessarily determined by a single vector of the mesoscale DMI. The *m*DMI is a result of the interplay between intrinsic spin-orbit- and extrinsic curvature-driven DMI terms and depends both on the material and geometrical parameters. We illustrated our approach on the example of a helix wire. By changing material and geometrical parameters we identified and investigated two stationary states: homogeneous quasitangential and periodical elliptic helicoidal states. Similarly to the case of straight wire with biaxial anisotropy, the periodical domain wall state appears near the boundary between two phases as a metastable state. We proposed an approach how these states could be verified in experiments based on integral and microscopic observations. The appearance of each state can be determined by measuring of the average values of the magnetization components and/or by establishing space Fourier spectra of the coordinate-dependent magnetic signals from nanohelices. Thus we propose a method to create new artificial chiral nanostructures with defined properties from standard magnetic materials by using geometrical manipulations^51,55–62^, which can be used in the development of the novel spintronics and spin-orbitronics devices. In this respect, the great development in nanotechnology, e.g. high-quality thin films growing approach, glancing angular deposition technology^58,62^, self-assembling methods and strain engineering techniques^56,57,59^, gives promise that these effects can be explored experimentally. It should be emphasized, that our model could be generalized for arbitrary 1D geometry and material parameters of experimentally obtained samples, by using proper parametrization with taking into account possible coordinate dependence of material parameters. More specifically, as the experimentally realized sculptured 3D cobalt nanowires do not have any intrinsic DMI^35^, our theory could be applied with ${{\mathcal{D}}}^{{\rm{I}}}=0$ and predicts the homogeneously magnetized quasitangential state for the same geometry (in the limit of ultrathin wire). Subsequent theoretical study of 1D helimagnetic nanowires require investigation of non-local magnetostatical effects, field-driven switching between states with different magnetic symmetry.

### Data availability

The datasets generated during and/or analysed during the current study are available from the corresponding author on reasonable request.

## Electronic supplementary material

Supplementary information
