# Supplementary material for: Mesoscale Dzyaloshinskii-Moriya interaction: geometrical tailoring of the magnetochirality
Source: Sci Rep. 2018 Jan 16;8:866. doi: 10.1038/s41598-017-18835-4 (PMC5770476; doi:10.1038/s41598-017-18835-4)
Supplement: Supplementary file 1 — Supplementary information [file 41598_2017_18835_MOESM1_ESM.pdf]

Mesoscale Dzyaloshinskii-Moriya interaction: geometrical tailoring of the magnetochirality

Oleksii M. Volkov[1,2*], **Denis D. Sheka**[3,†], **Yuri Gaididei**[2,**], **Volodymyr P. Kravchuk**[2,4,¶], **Ulrich K. Rößler**[4,Ø], **Jürgen Fassbender**[1,§], **and Denys Makarov**[1,‡]

[1]Helmholtz-Zentrum Dresden-Rossendorf e. V., Institute of Ion Beam Physics and Materials Research, 01328 Dresden, Germany
[2]Bogolyubov Institute for Theoretical Physics of the National Academy of Sciences of Ukraine, 03680 Kyiv, Ukraine
[3]Taras Shevchenko National University of Kyiv, 01601 Kyiv, Ukraine
[4]Leibniz-Institut für Festkörper- und Werkstoffforschung (IFW Dresden), D-01069 Dresden, Germany
[*]o.volkov@hzdr.de
[†]sheka@knu.ua
[‡]d.makarov@hzdr.de
[§]j.fassbender@hzdr.de
[Ø]u.roessler@ifw-dresden.de
[¶]vkravchuk@bitp.kiev.ua
[**]ybg@bitp.kiev.ua

ABSTRACT

The supplementary information provides details on analytical calculations of main aspects of the model of curvilinear magnetic wire with intrinsic Dzyaloshinskii-Moriya interaction and its spin-lattice simulations.

The model of curvilinear magnetic wire

Let us consider a curvilinear magnetic wire, which can be modelled by the 3D curve $\boldsymbol{\gamma}(s)$ with its natural arc length s parametrization. In the curvilinear systems it is convenient to use the Frenet-Serret references frame $\{\boldsymbol{e}_\text{T}, \boldsymbol{e}_\text{N}, \boldsymbol{e}_\text{B}\}$ with $\boldsymbol{e}_\text{T} = \partial_s \boldsymbol{\gamma}(s)$, $\boldsymbol{e}_\text{N} = \partial_{ss}\boldsymbol{\gamma}(s)/\kappa(s)$, and $\boldsymbol{e}_\text{B} = \boldsymbol{e}_\text{T} \times \boldsymbol{e}_\text{N}$ being the tangential, normal, and binormal unit vectors, respectively. Here ∂_s is derivative with respect to the natural parameter and $\kappa(s) = |\partial_{ss}\boldsymbol{\gamma}(s)|$ is the wire curvature. The relation between $\partial_s \boldsymbol{e}_\alpha$ and $\boldsymbol{e}_\alpha$ is determined by the Frenet-Serret formulas:

$$\partial_s \boldsymbol{e}_\alpha = F_{\alpha\beta}\boldsymbol{e}_\beta, \qquad \|F_{\alpha\beta}\| = \left\| \begin{smallmatrix} 0 & \kappa & 0 \\ -\kappa & 0 & \tau \\ 0 & -\tau & 0 \end{smallmatrix} \right\|, \tag{S1}$$

whereas Greek indices α, β enumerate curvilinear coordinates (TNB) and components of vector field, κ and τ are the curvature and torsion of the wire, respectively.

Our study is based on the Landau-Lifshitz phenomenological equation:[1,2]

$$\partial_t \boldsymbol{m} = \frac{\gamma_0}{M_s}\boldsymbol{m} \times \frac{\delta E}{\delta \boldsymbol{m}} + \eta\, \boldsymbol{m} \times \partial_t \boldsymbol{m}, \tag{S2}$$

where $\boldsymbol{m} = \boldsymbol{M}/M_s$ is the normalized magnetization vector, M_s is the saturation magnetization, ∂_t is derivative with respect to the time, γ_0 is the gyromagnetic ratio, η is a Gilbert damping constant and E is the total magnetic energy.

We consider here a narrow magnetic wire, the total energy $E = E_\text{an} + E_\text{ex} + E_\text{DMI}$ of which consists of three parts: exchange, anisotropic and intrinsic Dzyaloshinskii-Moriya interaction (DMI) contributions. The uniaxial anisotropy term reads

$$E_\text{an} = -KS \int \mathrm{d}s\, (\boldsymbol{m} \cdot \boldsymbol{e}_\text{an})^2, \tag{S3}$$

where S is the wire cross-section area, s is wire arc length, $K = K_0 + \pi M_s^2$ is the effective anisotropy constant, with $K_0 > 0$ being the magnetocrystalline anisotropy constant of easy-tangential type, the term πM_s^2 comes from the magnetostatic contribution.[3] The vector $\boldsymbol{e}_\text{an}$ in (S3) is the unit vector along the anisotropy axis, which is assumed to be oriented along the tangential direction of the curvilinear wire $\boldsymbol{e}_\text{an} = \boldsymbol{e}_\text{T}$.

The exchange energy has the form

$$E_{\rm ex} = -A\,S\int {\rm d}s\,\boldsymbol{m}\cdot\boldsymbol{\nabla}^2\boldsymbol{m} = -K\,S\,w^2\int {\rm d}s\,\boldsymbol{m}\cdot\boldsymbol{\nabla}^2\boldsymbol{m}, \tag{S4}$$

where A is an exchange constant and $w=\sqrt{A/K}$ is the characteristic magnetic length.

General form for the iDMI energy in the case of curvilinear magnetic system can be obtained from the atomic description of the DMI Hamiltonian:[4]

$$\mathcal{H}_{\rm DMI} = -\frac{1}{2}\sum_{(\boldsymbol{n},\boldsymbol{\delta})}\boldsymbol{d}_{\boldsymbol{n},\boldsymbol{n}+\boldsymbol{\delta}}\cdot\left[\boldsymbol{\mathcal{S}}_{\boldsymbol{n}}\times\boldsymbol{\mathcal{S}}_{\boldsymbol{n}+\boldsymbol{\delta}}\right], \tag{S5}$$

where $\boldsymbol{\mathcal{S}}_{\boldsymbol{n}}\equiv(\mathcal{S}_x,\mathcal{S}_y,\mathcal{S}_z)$ is a classical spin vector with fixed length $\mathcal{S}$ in units of action on the site $\boldsymbol{n}=(n_x,n_y,n_z)$ of a three–dimensional cubic lattice with integers n_x , n_y, n_z. The vector $\boldsymbol{\delta}$ connects nearest neighbors and $\boldsymbol{d}_{\boldsymbol{n},\boldsymbol{n}+\boldsymbol{\delta}}$ is the DMI vector for the bond $\boldsymbol{n},\boldsymbol{n}+\boldsymbol{\delta}$.

The direction of $\boldsymbol{d}_{\boldsymbol{n},\boldsymbol{n}+\boldsymbol{\delta}}$ depends on the crystallographic symmetry of the non-centrosymmetric materials: In the case of cubic crystals with T and O classes of symmetry, the vector $\boldsymbol{d}_{\boldsymbol{n},\boldsymbol{n}+\boldsymbol{\delta}}$ is aligned in the same direction as the vector $\boldsymbol{\xi}_{\boldsymbol{n},\boldsymbol{n}+\boldsymbol{\delta}}$,[4] which is the unit vector between sites $\boldsymbol{n}$ an $\boldsymbol{n}+\boldsymbol{\delta}$; in the case of other classes of crystal symmetry, the vector $\boldsymbol{d}_{\boldsymbol{n},\boldsymbol{n}+\boldsymbol{\delta}}$ is tilted from the vector $\boldsymbol{\xi}_{\boldsymbol{n},\boldsymbol{n}+\boldsymbol{\delta}}$ by some angle, which can even reach the value $\pi/2$ for ultrathin magnetic layers on heavy metal of the adjacent layer.[5,6]

The continuum dynamics of the spin system can be described in terms of magnetization unit vector $\boldsymbol{m}$ and due to the assumption that spin direction evolves slowly at the atomic scale, one can derive the DMI energy term for the arbitrary one-dimensional curvilinear magnetic system:

$$E_{\rm DMI} = -S\int {\rm d}s\boldsymbol{D}^{\rm I}\cdot\left[\boldsymbol{m}\times(\boldsymbol{e}_{\rm T}\cdot\boldsymbol{\nabla})\boldsymbol{m}\right] = -S\int {\rm d}s\boldsymbol{D}^{\rm I}\cdot\left[\boldsymbol{m}\times\partial_s\boldsymbol{m}\right] = -K\,S\int {\rm d}\xi\,\boldsymbol{\mathfrak{D}}^{\rm I}\cdot\left[\boldsymbol{m}\times\boldsymbol{m}'\right], \tag{S6}$$

where $\boldsymbol{D}^{\rm I}$ is the DMI vector with the continuous DMI constant $D^{\rm I}$. The link between $D^{\rm I}$ and d depends on the lattice type, but it scales with $d\,\mathcal{S}^2/a^2$ with a being the lattice constant. In (S6) prime denotes the derivative with respect to the dimensionless coordinate $\xi=s/w$, the $\boldsymbol{\mathfrak{D}}^{\rm I}=\boldsymbol{D}/\sqrt{AK}$ is the reduced vector of the iDMI.

Using the TNB-parametrization of the magnetization $\boldsymbol{m}=m_{\rm T}\boldsymbol{e}_{\rm T}+m_{\rm N}\boldsymbol{e}_{\rm N}+m_{\rm B}\boldsymbol{e}_{\rm B}$, one can represent the exchange energy as follows:[7]

$$E_{\rm ex} = K\,S\int {\rm d}\xi\;\mathscr{E}_{\rm ex}, \qquad \mathscr{E}_{\rm ex} = \left|\boldsymbol{m}'\right|^2 + \mathscr{F}_{\alpha\beta}\left(m_\alpha m'_\beta - m'_\alpha m_\beta\right) + \mathscr{K}_{\alpha\beta}\,m_\alpha m_\beta, \tag{S7}$$

where the Einstein notation is used for summation. The first term is the isotropic part of the exchange expression and it has formally the same form as for a straight wire. The second term in (S7) is the chiral term, which has the form of the set of the Lifshitz invariants and describe the geometry symmetry braking. This set of the Lifshitz invariants in the curvilinear frame of reference can be referred to a curvature-induced DMI, which is linear with respect to the reduced curvature $\varkappa=\kappa w$ and torsion $\sigma=\tau w$. The tensor $\mathscr{F}_{\alpha\beta}=wF_{\alpha\beta}$ is the dimensionless Frenet-Serret tensor

$$\left\|\mathscr{F}_{\alpha\beta}\right\| = \left\|\begin{matrix} 0 & \varkappa & 0\\ -\varkappa & 0 & \sigma\\ 0 & -\sigma & 0\end{matrix}\right\|.$$

The third term in (S7) describes an effective curvature-induced anisotropy, where the components of the tensor $\mathscr{K}_{\alpha\beta}=\mathscr{F}_{\alpha\nu}\mathscr{F}_{\beta\nu}$, the components of which are bilinear with respect to $\varkappa$ and σ:

$$\left\|\mathscr{K}_{\alpha\beta}\right\| = \left\|\begin{matrix} \varkappa^2 & 0 & -\varkappa\sigma\\ 0 & \varkappa^2+\sigma^2 & 0\\ -\varkappa\sigma & 0 & \sigma^2\end{matrix}\right\|.$$

It is also possible to rewrite the exchange energy expression (S7) in the following form:

$$\mathscr{E}_{\rm ex} = \left|\boldsymbol{m}'\right|^2 - \boldsymbol{\mathfrak{D}}^{\rm E}\cdot\left[\boldsymbol{m}\times\boldsymbol{m}'\right] + \left(\sigma m_{\rm T} + \varkappa m_{\rm B}\right)^2, \tag{S8}$$

due to the use of the Frenet-Serret formulas and the property $|\boldsymbol{m}|=1$. In the (S8) $\boldsymbol{\mathfrak{D}}^{\rm E}=-2\sigma\boldsymbol{e}_{\rm T}-2\varkappa\boldsymbol{e}_{\rm B}$ denotes the reduced vector of the extrinsic curvature-driven DMI.

Using the Frenet-Serret formulas one can write the density of the total energy in the form:

$$\mathscr{E} = \left|\boldsymbol{m}'\right|^2 + \mathscr{D}^{\rm meso}_{\alpha\beta}\left(m_\alpha m'_\beta - m'_\alpha m_\beta\right) + \mathscr{K}^{\rm meso}_{\alpha\beta}\,m_\alpha m_\beta, \tag{S9}$$

where explicit forms of the effective anisotropy and DMI tensors, respectively, $\mathscr{D}^{\text{eff}}_{\alpha\beta}$ and $\mathscr{K}^{\text{eff}}_{\alpha\beta}$, read

$$\left\|\mathscr{D}^{\text{meso}}_{\alpha\beta}\right\| = \left\|\begin{matrix} 0 & -(\mathfrak{D}^{\text{I}}_{\text{B}}/2-\varkappa) & -\mathfrak{D}^{\text{I}}_{\text{N}}/2 \\ \mathfrak{D}^{\text{I}}_{\text{B}}/2-\varkappa & 0 & -(\mathfrak{D}^{\text{I}}_{\text{T}}/2-\sigma) \\ \mathfrak{D}^{\text{I}}_{\text{N}}/2 & \mathfrak{D}^{\text{I}}_{\text{T}}/2-\sigma & 0 \end{matrix}\right\|, \tag{2b}$$

$$\left\|\mathscr{K}^{\text{meso}}_{\alpha\beta}\right\| = \left\|\begin{matrix} \sigma(\mathfrak{D}^{\text{I}}_{\text{T}}-\sigma)-1 & \mathfrak{D}^{\text{I}}_{\text{N}}\sigma/2 & [\varkappa(\mathfrak{D}^{\text{I}}_{\text{T}}-\sigma)+\sigma(\mathfrak{D}^{\text{I}}_{\text{B}}-\varkappa)]/2 \\ \mathfrak{D}^{\text{I}}_{\text{N}}\sigma/2 & 0 & \mathfrak{D}^{\text{I}}_{\text{N}}\varkappa/2 \\ [\varkappa(\mathfrak{D}^{\text{I}}_{\text{T}}-\sigma)+\sigma(\mathfrak{D}^{\text{I}}_{\text{B}}-\varkappa)]/2 & \mathfrak{D}^{\text{I}}_{\text{N}}\varkappa/2 & \varkappa(\mathfrak{D}^{\text{I}}_{\text{B}}-\varkappa) \end{matrix}\right\| \tag{2c}$$

due to the use of the property $|\boldsymbol{m}| = 1$.

Rotated frame of references

In the following we consider that the vector of the intrinsic DMI $\mathfrak{D}$ is directed along the tangential direction of a curvilinear wire. As it was shown in (2), the tensor of the effective anisotropy coefficients $\mathscr{K}^{\text{meso}}_{\alpha\beta}$ has a non-diagonal components, which is typical for the biaxial magnets and means that the homogeneous magnetization state is not oriented along one fixed axis of the TNB basis. One can easily diagonalize it, by using a unitary transformation (rotation in a local rectifying plane) of the vector $\boldsymbol{m}$

$$\boldsymbol{m}^T = U\widetilde{\boldsymbol{m}}^T, \qquad \widetilde{\boldsymbol{m}}^T = U^{-1}\boldsymbol{m}^T, \qquad \widetilde{\boldsymbol{m}} = m_1\boldsymbol{e}_1 + m_2\boldsymbol{e}_2 + m_3\boldsymbol{e}_3,, \qquad U = \left\|\begin{matrix} \cos\psi & 0 & -\sin\psi \\ 0 & 1 & 0 \\ \sin\psi & 0 & \cos\psi \end{matrix}\right\|. \tag{S10}$$

By choosing the rotation angle ψ as follows

$$\begin{aligned} \psi &= \arctan\frac{\varkappa(\sigma-\mathfrak{D}^{\text{I}}_{\text{T}}/2)}{\mathscr{K}_0}, \\ \mathscr{K}_0 &= \frac{1+\sigma^2-\varkappa^2-\mathfrak{D}^{\text{I}}_{\text{T}}\sigma+\mathscr{K}_1}{2}, \\ \mathscr{K}_1 &= \sqrt{(1-\varkappa^2+\sigma^2-\mathfrak{D}^{\text{I}}_{\text{T}}\sigma)^2+(2\sigma-\mathfrak{D}^{\text{I}}_{\text{T}})^2\varkappa^2}, \end{aligned} \tag{S11}$$

one can easily reduce the anisotropy energy $\mathscr{E}^{\text{A}}_{\text{eff}}$ to the form

$$\begin{aligned} \mathscr{E}^{\text{A}}_{\text{eff}} &= -\mathscr{K}_1 m_1^2 + \mathscr{K}_2 m_2^2, \\ \mathscr{K}_2 &= \frac{1+\varkappa^2+\sigma^2-\mathfrak{D}^{\text{I}}_{\text{T}}\sigma-\mathscr{K}_1}{2}. \end{aligned} \tag{S12}$$

In the same way one can derive the effective DMI energy terms in the new frame of reference:

$$\begin{aligned} \mathscr{E}^{\text{D}}_{\text{ex}} &= \mathscr{D}_1\left(m_2m_3'-m_3m_2'\right)+\mathscr{D}_2\left(m_1m_2'-m_2m_1'\right), \\ \mathscr{D}_1 &= 2\left(\sigma-\frac{\mathfrak{D}^{\text{I}}_{\text{T}}}{2}\right)\frac{\mathscr{K}_0+\varkappa^2}{\sqrt{\mathscr{K}_0^2+\varkappa^2(\sigma-\mathfrak{D}^{\text{I}}_{\text{T}}/2)^2}}, \\ \mathscr{D}_2 &= 2\varkappa\frac{\mathscr{K}_0-(\sigma-\mathfrak{D}^{\text{I}}_{\text{T}}/2)^2}{\sqrt{\mathscr{K}_0^2+\varkappa^2(\sigma-\mathfrak{D}^{\text{I}}_{\text{T}}/2)^2}}. \end{aligned} \tag{S13}$$

The coefficient $\mathscr{K}_1$ characterizes the strength of the effective easy-axis anisotropy while $\mathscr{K}_2$ gives the strength of the effective easy-surface anisotropy. The parameters $\mathscr{D}_1$ and $\mathscr{D}_2$ are the effective mDMI constants, which are responsible for two types of magnetization rotation: around the direction $\boldsymbol{e}_1$ and $\boldsymbol{e}_3$, respectively. The directions of the rotated ψ-frame $\{\boldsymbol{e}_1,\boldsymbol{e}_2,\boldsymbol{e}_3\}$ have the following form:

$$\boldsymbol{e}_1 = \boldsymbol{e}_{\text{T}}\cos\psi+\boldsymbol{e}_{\text{B}}\sin\psi, \qquad \boldsymbol{e}_2 = \boldsymbol{e}_{\text{N}}, \qquad \boldsymbol{e}_3 = -\boldsymbol{e}_{\text{T}}\sin\psi+\boldsymbol{e}_{\text{B}}\cos\psi. \tag{S14}$$

Cycloidal state

As a specific case of the general elliptical helicoidal state in a helix wire, we treat analytically cycloidal state in the rotated frame of references. It is realized under the condition $\sigma = \sigma_0 = \mathfrak{D}^{\text{I}}_{\text{T}}/2$ and corresponds to the situation when the vector of mDMI is aligned along the hard axis $\boldsymbol{e}_3$. The effective anisotropy and DMI constants read

$$\begin{aligned} \mathscr{K}^{\text{cy}}_1 &= \left|1-\varkappa^2-(\mathfrak{D}^{\text{I}}_{\text{T}})^2/4\right|, \\ \mathscr{K}^{\text{cy}}_2 &= \frac{1+\varkappa^2-(\mathfrak{D}^{\text{I}}_{\text{T}})^2/4-\mathscr{K}^{\text{cy}}_1}{2}, \\ \mathscr{D}^{\text{cy}}_1 &= 0, \\ \mathscr{D}^{\text{cy}}_2 &= 2\varkappa. \end{aligned} \tag{S15}$$

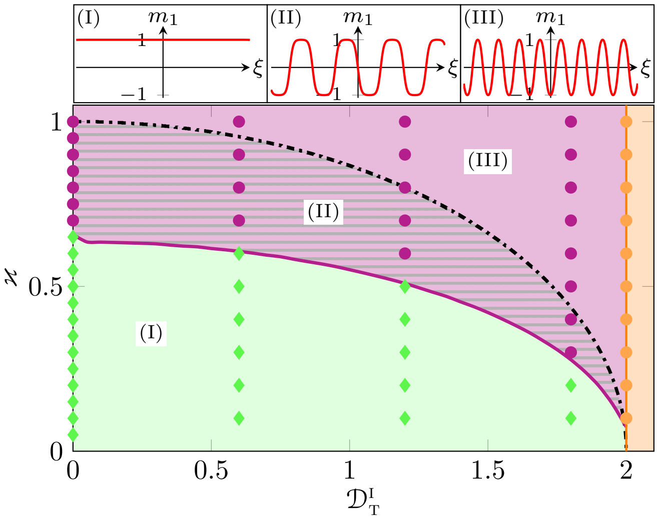

Figure S1. Phase diagrams for $\sigma = \mathfrak{D}_{\mathrm{T}}^{\mathrm{I}}/2$, which shows the transition from quasitangential to the cycloidal state. The inset pictures (I), (II) and (III) correspond to the schematic magnetic distributions of quasitangential, periodical domain wall and cycloidal states. Dashed line represents the dependence of $\varkappa_c$ for domain wall instability.

In order to derive the exact solution of the cycloidal state in the rotated ψ–frame, it is convenient to use an angular parametrization $\widetilde{\boldsymbol{m}} = \{\sin\theta\cos\phi, \sin\theta\sin\phi, \cos\theta\}$. The minimization of the energy density (5) with (S15) results in $\theta^{\mathrm{cy}} = \pi/2$ and the azimuthal angle $\phi^{\mathrm{cy}} = \pi/2 - \Phi$, where Φ satisfies the pendulum equation:[7]

$$\Phi'' + \left(\mathscr{K}_1^{\mathrm{cy}} + \mathscr{K}_2^{\mathrm{cy}}\right)\sin\Phi\cos\Phi = 0. \tag{S16}$$

The inhomogeneous solution of this equation reads

$$\theta^{\mathrm{cy}} = \frac{\pi}{2}, \qquad \phi^{\mathrm{cy}} = \frac{\pi}{2} - \mathrm{am}\left(\zeta^{\mathrm{cy}}, k^{\mathrm{cy}}\right), \qquad \zeta^{\mathrm{cy}} = \frac{\xi}{k^{\mathrm{cy}}}\sqrt{\mathscr{K}_1^{\mathrm{cy}} + \mathscr{K}_2^{\mathrm{cy}}}, \tag{S17}$$

where $\mathrm{am}(\zeta, k)$ is Jacobi's amplitude[8] and the modulus k^{cy} is determined by the condition

$$\lambda^{\mathrm{cy}}\sqrt{\mathscr{K}_1^{\mathrm{cy}} + \mathscr{K}_2^{\mathrm{cy}}} = 4k^{\mathrm{cy}}\,\mathrm{K}(k^{\mathrm{cy}}), \tag{S18}$$

with λ^{cy} being a period of cycloidal modulations and $\mathrm{K}(k)$ being the complete elliptic integral of the first kind.[8] The energy density of this state reads

$$\mathscr{E}^{\mathrm{cy}} = \mathscr{K}_1^{\mathrm{cy}} + \frac{\mathscr{K}_1^{\mathrm{cy}} + \mathscr{K}_2^{\mathrm{cy}}}{(k^{\mathrm{cy}})^2}\left[(1+k^{\mathrm{cy}})\,\mathrm{dn}^2(\zeta^{\mathrm{cy}}, k^{\mathrm{cy}}) - k^{\mathrm{cy}}\frac{\mathscr{D}_2^{\mathrm{cy}}}{\sqrt{\mathscr{K}_1^{\mathrm{cy}} + \mathscr{K}_2^{\mathrm{cy}}}}\,\mathrm{dn}(\zeta^{\mathrm{cy}}, k^{\mathrm{cy}}) - k^{\mathrm{cy}}\right], \tag{S19}$$

where $\mathrm{dn}(\zeta, k)$ is the Jacobi's elliptic function.[8] Integrating the energy density over one period of the modulations and minimizing with respect to the period λ^{cy} leads to the exact form of the energy of cycloid modulations E^{cy}. The equality of energies $E^{\mathrm{qt}}(\varkappa, \mathfrak{D}_{\mathrm{T}}^{\mathrm{I}}) = E^{\mathrm{cy}}(\varkappa, \mathfrak{D}_{\mathrm{T}}^{\mathrm{I}})$ determines the boundary curve, which separates the quasitangential and the cycloidal states, Fig. 2 (g1) ($\sigma = \mathfrak{D}_{\mathrm{T}}^{\mathrm{I}}/2$ line) and solid magenta line on the phase diagram Fig. S1 (a).

Spin-lattice simulations

We verify our predictions about the mDMI and its influence on the magnetic system by performing three-dimensional spin-lattice simulations using in-house developed program SLaSi.[9] We model the ferromagnetic helix by considering a classical chain of magnetic moments $\boldsymbol{m}_i$, with $i = \overline{1,N}$, situated on a helix. The system is described by the set of N discrete Landau-Lifshitz-Gilbert equations,

$$\frac{\mathrm{d}\boldsymbol{m}_i}{\mathrm{d}t} = \omega_0\,\boldsymbol{m}_i \times \frac{\partial \mathcal{E}}{\partial \boldsymbol{m}_i} + \eta\,\boldsymbol{m}_i \times \frac{\mathrm{d}\boldsymbol{m}_i}{\mathrm{d}t}, \tag{S20}$$

where $\omega_0 = 4\pi\gamma_0 M_s$ and $\mathcal{E} = \mathcal{E}_{\text{ex}} + \mathcal{E}_{\text{an}} + \mathcal{E}_{\text{dmi}}$ is a dimensionless magnetic energy of the helix wire, normalized by $4\pi M_s^2 a^2$ with a being the lattice constant. In our simulation we consider the exchange exchange energy of the magnetic system,

$$\mathcal{E}_{\text{ex}} = -2\frac{\ell^2}{a^2}\sum_{i=1}^{N-1} \boldsymbol{m}_i \cdot \boldsymbol{m}_{i+1}, \tag{S21a}$$

where $\ell = \sqrt{A/(4\pi M_s^2)}$ is an exchange length. The easy-tangential anisotropy term,

$$\mathcal{E}_{\text{an}} = -\frac{Q}{2}\sum_{i=1}^{N-1} (\boldsymbol{m}_i \cdot \boldsymbol{u}_{i,i+1})^2, \tag{S21b}$$

where $Q = K/(2\pi M_s^2)$ is a quality factor and $\boldsymbol{u}_{i,j}$ is the unit vector which connects i-th and j-th sites. And the iDMI term,

$$\mathcal{E}_{\text{dmi}} = -\frac{\mathcal{D}_{\text{T}}^{\text{I}} Q}{4a}\sum_{i=1}^{N-1} \boldsymbol{u}_{i,i+1} \cdot [\boldsymbol{m}_i \times \boldsymbol{m}_{i+1}], \tag{S21c}$$

where it was estimated that the DMI vector is aligned parallel to $\boldsymbol{u}_{i,i+1}$.

To study the equilibrium magnetization states we consider a chain of $N = 1000$ sites. In all simulations the magnetic length $w = 15a$. The curvature and torsion of the helix wire are varied in the wide range of parameters, respectively, $\varkappa \in (0,1)$ and $\sigma \in (-1,3)$, with the step $\Delta\varkappa = \Delta\sigma = 0.1$. The reduced value of the iDMI constant $\mathcal{D}_{\text{T}}^{\text{I}}$ is varied in the range $\mathcal{D}_{\text{T}}^{\text{I}} \in (0,3)$ with the step 0.3.

In order to verify our analytical predictions, we perform our simulations starting from different initial magnetization distribution, namely: uniform magnetization state in the Cartesian frame of reference, which aligns along the $\hat{\boldsymbol{z}}$ direction; periodical magnetization states in the Cartesian frame of reference, which align along the $\boldsymbol{e}_{\text{T}}$ and $\boldsymbol{e}_{\text{N}}$ directions in the TNB frame; and quasitangential magnetization state, which align along the $\boldsymbol{e}_1$ directions in the rotated curvilinear ψ–frame. We simulate numerically the set of N discrete Landau-Lifshitz-Gilbert equations (S20) in the overdamped regime with damping coefficient $\eta = 0.5$ during the long-time interval $\Delta t \gg (\eta\,\omega_0)^{-1}$. The final state with the lowest energy is considered to be the equilibrium magnetization state. Simulations data are presented on the Fig.2 (g) by filled symbols together with theoretical results. One can see a good correspondence between simulations and our analytical results.

All simulations for this work were performed using the computer clusters of the Bayreuth University,[10] Taras Schevchenko National University of Kyiv[11] and Bogolyubov Institute for Theoretical Physics of the National Academy of Sciences of Ukraine.[12]

References

1. Landau, L. D. & Lifshitz, E. M. On the theory of the dispersion of magnetic permeability in ferromagnetic bodies. *Phys. Zs. Sowjet.; Reproduces in in Ukr. J. Phys. 53 25-35 (2008)* **8**, 153–169 (1935). URL http://ujp.bitp.kiev.ua/files/journals/53/si/53SI08p.pdf.

2. Gilbert, T. A phenomenological theory of damping in ferromagnetic materials. *IEEE Transactions on Magnetics* **40**, 3443–3449 (2004). URL http://dx.doi.org/10.1109/TMAG.2004.836740.

3. Slastikov, V. V. & Sonnenberg, C. Reduced models for ferromagnetic nanowires. *IMA Journal of Applied Mathematics* **77**, 220–235 (2012). URL http://dx.doi.org/10.1093/imamat/hxr019.

4. Moriya, T. Anisotropic superexchange interaction and weak ferromagnetism. *Phys. Rev.* **120**, 91–98 (1960). URL http://link.aps.org/abstract/PR/v120/p91.

5. Fert, A. Magnetic and transport properties of metallic multilayers. *Materials Science Forum* **59-60**, 439–480 (1990). URL http://dx.doi.org/10.4028/www.scientific.net/MSF.59-60.439.

6. Rohart, S. & Thiaville, A. Skyrmion confinement in ultrathin film nanostructures in the presence of Dzyaloshinskii-Moriya interaction. *Physical Review B* **88**, 184422 (2013). URL http://dx.doi.org/10.1103/PhysRevB.88.184422.

7. Sheka, D. D., Kravchuk, V. P. & Gaididei, Y. Curvature effects in statics and dynamics of low dimensional magnets. *Journal of Physics A: Mathematical and Theoretical* **48**, 125202 (2015). URL http://stacks.iop.org/1751-8121/48/i=12/a=125202.

8. Olver, F. W. J., Lozier, D. W., Boisvert, R. F. & Clark, C. W. (eds.) *NIST Handbook of Mathematical Functions* (Cambridge University Press, New York, NY, 2010). URL http://www.cambridge.org/us/academic/subjects/mathematics/abstract-analysis/nist-handbook-mathematical-functions.

9. SLaSi spin–lattice simulations package. http://slasi.knu.ua. URL http://slasi.knu.ua. Accessed: 2016-04-17.

10. Computing center of bayreuth university. URL http://www.rz.uni-bayreuth.de.

11. High–performance computing cluster of Taras Shevchenko National University of Kyiv. URL http://cluster.univ.kiev.ua/eng/.

12. Computing grid-cluster of the Bogolyubov Insitute for Theoretical Physics of NAS of Ukraine. URL http://horst-7.bitp.kiev.ua.
